# Tuberculosis Drug Resistance and HIV Infection, the Netherlands

**DOI:** 10.3201/eid1305.060334

**Published:** 2007-05

**Authors:** Catharina Hendrika Haar, Frank G.J. Cobelens, Nico A. Kalisvaart, Jan J. van der Have, Paul J.H.J. van Gerven, Dick van Soolingen

**Affiliations:** * KNCV Tuberculosis Foundation, The Hague, the Netherlands; † Municipal Health Service, Groningen, the Netherlands; ‡ Academic Medial Centre, Amsterdam, the Netherlands; § National Institute of Public Health and the Environment, Bilthoven, the Netherlands

**Keywords:** HIV, tuberculosis, drug resistance, multidrug resistance, dispatch

## Abstract

In the Netherlands during 1993–2001, multidrug-resistant tuberculosis among newly diagnosed patients was more frequent in those with HIV coinfection (5/308, 1.6%) than in those with no HIV infection (39/646, 0.6%; adjusted odds ratio 3.43, p = 0.015). Four of the 5 patients coinfected with multidrug-resistant tuberculosis and HIV were foreign-born. DNA fingerprint analysis suggested that transmission had occurred outside the Netherlands.

HIV infection strongly increases the risk for tuberculosis (TB) infection: TB disease occurs in 7%–10% of patients with HIV infection each year (*1*). The increase in numbers of patients with both HIV infection and TB has raised the potential for increasing transmission of drug-resistant *Mycobacterium tuberculosis* strains (*2*).

Reports on associations of HIV coinfection and drug resistance among patients with TB have been contradictory. Some studies found strongly increased risks for multidrug-resistant TB (MDR TB) among patients coinfected with TB and HIV (*3*–*7*), whereas other studies found no increased risk (*8*–*11*). Population-based data are limited, however, in particular from low-prevalence countries. We report on a population-based study of anti-tuberculosis resistance patterns and associations with HIV infection in the Netherlands during 1993–2001.

## The Study

Patient data were obtained from the Netherlands Tuberculosis Register (NTR), which contains data on all TB cases since 1993 reported by TB control departments of municipal health services. Data on drug susceptibility were obtained from the National Tuberculosis Reference Laboratory (National Institute of Public Health and the Environment, Bilthoven, the Netherlands), which performs drug-susceptibility testing (DST) and restriction fragment length polymorphism (RFLP) typing on all *M. tuberculosis* complex strains isolated from patients in the Netherlands. DST is performed according to an absolute concentration method on 7H10 agar, with a proportional cut-off (*12*). RFLP typing is based on the standardized method (*13*). Laboratory records were matched to NTR records by a combination of postal code, date of birth, and sex.

In the NTR, HIV infection is recorded as a response option on the item on impaired immunity. Patients with a record of impaired immunity due to HIV infection were considered HIV positive. If immunity was reported to be impaired because of other causes than HIV infection, or if immune status was unknown, HIV status was considered negative. If the item on impaired immunity was missing (not filled out), the record was excluded from the analysis.

In the analysis, the association was determined between HIV status and resistance to various drugs and combinations of drugs used to treat TB. MDR TB was defined as resistance of *M. tuberculosis* to at least isoniazid and rifampin. DST results before start of treatment were included unless cultures had only been taken during treatment. Patients were categorized as either previously treated or new (i.e., previously untreated). Previous treatment was defined as a history of TB treatment for >4 weeks, a sputum sample obtained during treatment, or both.

For comparison of categorical variables, significance testing was done by χ^2^ test with continuity correction or by 2-sided Fisher exact test as appropriate. Multivariate analysis was conducted by logistic regression. A p value <0.05 was considered statistically significant unless stated otherwise. Analyses were conducted with SPSS version 12.0.1 (SPSS Inc., Chicago, IL, USA).

During the study period, 13,943 TB cases were reported to NTR (57% in foreign-born patients), including 8,450 persons with positive *M. tuberculosis* cultures. Of the case-patients with positive cultures, 7,354 were identified in the laboratory database (87.0%). Excluded were 264 (3.6%) of 7,354 case-patients because of missing information on impaired immunity, which left 7,090 case-patients for the analysis. No significant differences between included and excluded case-patients were found in age, sex, nationality, localization of disease, place of residence, risk groups, or year of diagnosis. More included than excluded case-patients had received a TB diagnosis in a hospital, 5,500 (78%) versus 4,310 (62%), respectively (p = 0.034).

HIV infection was reported in 329 (4.6%) of the 7,090 included case-patients. Of these, 232 (71%) were male, 198 (60%) were of foreign origin, 118 (36%) were of Dutch origin, and 13 (4%) were of unknown origin. The 198 case-patients of foreign origin included 128 (41%) patients from Africa and 24 (8%) from industrialized countries; the remaining 49 (14%) patients were from Asia and Central and South America

Of the 7,090 case-patients included in the study, 6,775 (95.6%) were new and 315 (4.4%) were previously treated. Among the new cases, drug resistance was reported in 817 (12.1%); isoniazid resistance was reported in 449 (6.6%), rifampin resistance in 51 (0.8%), and multidrug resistance in 44 (0.7%). Multidrug resistance was significantly associated with HIV infection both before (odds ratio [OR] 2.78, p = 0.033) and after adjustment by multivariate analysis for age, sex, and continent of origin (adjusted OR 3.43, p = 0.015). In addition, near-significant associations with HIV infection were observed for resistance to isoniazid (OR 1.50, 95% confidence interval [CI] 0.99–2.26) and resistance to rifampin (OR 2.35, 95% CI 0.82–6.24) (Table 1).

**Table 1:** Association between HIV infection and primary drug resistance among new tuberculosis patients, the Netherlands, 1993–2001*

	No. HIV negative (%) (n = 6,467)	No. HIV positive (%) (n = 308)	OR (95% CI) (unadjusted)	p value†
Fully susceptible	5,695 (88.1)	263 (85.4)	1.00	
Resistant to 1 drug	544 (8.4)	29 (9.4)	1.15 (0.76–1.74)	0.542
Resistant to 2 drugs	193 (3.0)	11 (3.6)	1.23 (0.63–2.36)	0.622
Resistant to 3 drugs	24 (0.4)	5 (1.6)	4.51 (1.50–12.57)	0.001
Resistant to 4 drugs	11 (0.2)	0	–	–
Any resistance	772 (11.9)	45 (17.1)	1.26 (0.90–1.77)	0.188
Any resistance to:				
Isoniazid	420 (6.5)	29 (9.4)	1.50 (0.99–2.26)	0.059
Rifampin	46 (0.7)	5 (1.6)	2.35 (0.82–6.24)	0.075
Streptomycin	538 (8.3)	31 (10.1)	1.25 (0.83–1.86)	0.303
Ethambutol	42 (0.6)	1 (0.3)	0.52 (0.03–3.49)	1.000
Multidrug resistance‡	39 (0.6)	5 (1.6)	2.78 (1.09–7.10)	0.033

*New patients are defined as those not previously treated for tuberculosis. OR, odds ratio; CI, confidence interval. †p value determined by Fisher exact test or χ^2^ test (Yates corrected), as appropriate. ‡Resistant to at least isoniazid and rifampin.

Among the 315 previously treated patients, drug resistance was reported in 68 (21.6%); isoniazid resistance was reported in 52 (16.5%), rifampin resistance in 19 (6.0%), and multidrug resistance in 17 (5.4%). HIV infection was significantly associated with any rifampin resistance (OR 4.12, CI 1.01–15.67, p<0.05) (Table 2). Monoresistance to rifampin was found in 2 previously treated patients; both were HIV infected.

**Table 2:** Prevalence of secondary drug resistance among previously treated tuberculosis patients, the Netherlands, 1993–2001*

	No. HIV negative (%) (n = 294)	No. HIV positive (%) (n = 21)
Fully susceptible	232 (78.9)	15 (71.4)
Resistant to 1 drug: Isoniazid Rifampin Streptomycin	34 (11.9) 20 (6.8) 0 14 (4.8)	4 (19.0) 2 (9.5) 2 (9.5) 0
Resistant to 2 drugs	17 (5.8)	0
Resistant to 3 drugs	4 (1.4)	0
Resistant to 4 drugs	7 (2.4)	2 (9.5)
Any resistance	62 (21.1)	6 (28.6)
Any resistance to:		
Isoniazid	48 (16.3)	4 (19.0)
Rifampin	15 (5.1)	4 (19.0)*
Streptomycin	36 (12.2)	2 (9.5)
Ethambutol	9 (3.1)	2 (9.5)
Multidrug resistance†	15 (5.1)	2 (9.5)

*Undajusted odds ratio 4.12 (95% confidence interval 1.01–15.67, p = 0.036). †Resistant to at least isoniazid and rifampin.

The 5 new HIV-infected MDR TB patients (4 men, 1 woman; age range 22–31 years) originated from the Netherlands, Liberia, Angola, South Africa, and Portugal. Four had diagnoses of pulmonary TB and 1 extrapulmonary TB. None had a known history of intravenous drug use. Two patients completed treatment, 2 died during treatment, and 1 continued treatment at an unknown location.

The 2 previously treated HIV-infected MDR TB patients (both women, ages 28 and 43 years) originated from Europe. Pulmonary TB was diagnosed for both; 1 was an intravenous drug user. One died during treatment, and the other was lost to follow-up.

Each of the 7 patients with both MDR TB and HIV infection had different RFLP patterns. Four of these patients, all new, shared an RFLP pattern with >1 other patient in the database. For 1 of these, the cluster included other MDR TB patients. Transmission could have occurred from this patient with MDR TB and HIV infection to 2 other patients without HIV infection whose TB had been diagnosed in the same year. The same patient, whose infecting strain was resistant to isoniazid, rifampin, and streptomycin, could have acquired the TB infection in the Netherlands from an African patient with HIV infection who had received a diagnosis of TB 3 years earlier and harbored a strain resistant to isoniazid and streptomycin. For the other 3 clustered patients, the resistance patterns or date of entry into the country made transmission to or from other patients within the Netherlands impossible.

In the 2 cases of monoresistance of TB to rifampin, the isolates were obtained before treatment from patients with HIV infection originating from Cape Verde and Somalia. Both had combined pulmonary and extrapulmonary TB and had been treated before in the Netherlands. One had completed 6 months of treatment; for the other, treatment completion was not recorded.

## Conclusions

Overall, MDR TB occurred in 5 (2.5%) of 198 foreign-born patients with TB and HIV infection, compared with 2 (1.7%) of 118 Dutch-born patients with TB and HIV coinfection (p = 0.730). We found low prevalence of multidrug resistance among patients with TB, in accordance with an earlier study that covered a shorter period (*8*). In the Netherlands, transmission of MDR TB has been rare. During the study period, single secondary MDR TB cases, as confirmed by RFLP typing, were documented in only 2 instances, 1 nosocomial (M. Sebek, pers. comm.). This may be related to the presence of a system of drug resistance surveillance with national coverage, active contact tracing around infectious TB cases, and directly observed treatment of patients with TB in specialized centers under strict respiratory isolation.

Only 7 cases of MDR TB occurred among 329 patients with HIV infection during this 9-year period (2.1%). Despite this small number, MDR TB was significantly more frequent among previously untreated patients with TB and HIV infection than among those without HIV infection. Even though the results were adjusted, at least partially, for origin of the patient, non-Dutch origin appears to play an important role in this association. Of the 5 patients with new TB and HIV infection, 4 were foreign-born, including 3 from sub-Saharan Africa. Because transmission in the Netherlands could be ruled out in 4 of the 5 new cases, most if not all of these infections were acquired abroad. The MDR TB infections in these patients may have been acquired in institutional settings such as hospitals, but data on the pre-immigration history were lacking.

Four (19%) of 21 case-patients with previously treated TB and HIV infection had rifampin-resistant isolates, including 2 (10%) that were rifampin monoresistant. Acquisition of monoresistance to rifampin is associated with HIV infection and may be related to intestinal malabsorption, intermittent treatment with rifabutin, and drug interactions (*14*,*15*). In the patients in our study, the contribution of these factors could not be established.

In conclusion, among new TB patients in the Netherlands, multidrug resistance is associated with HIV infection, predominantly as an imported disease. In patients with HIV infection who have previously been treated for TB, the possibility of rifampin resistance should be considered. Routine surveillance of resistance to anti-TB drugs will improve timely recognition of MDR TB cases and help prevent further transmission.
